# Supplementary figures and images for: Transcriptomic profiling in canines and humans reveals cancer specific gene modules and biological mechanisms common to both species
Source: PLoS Comput Biol. 2021 Sep 27;17(9):e1009450. doi: 10.1371/journal.pcbi.1009450 (PMC8523068; doi:10.1371/journal.pcbi.1009450)

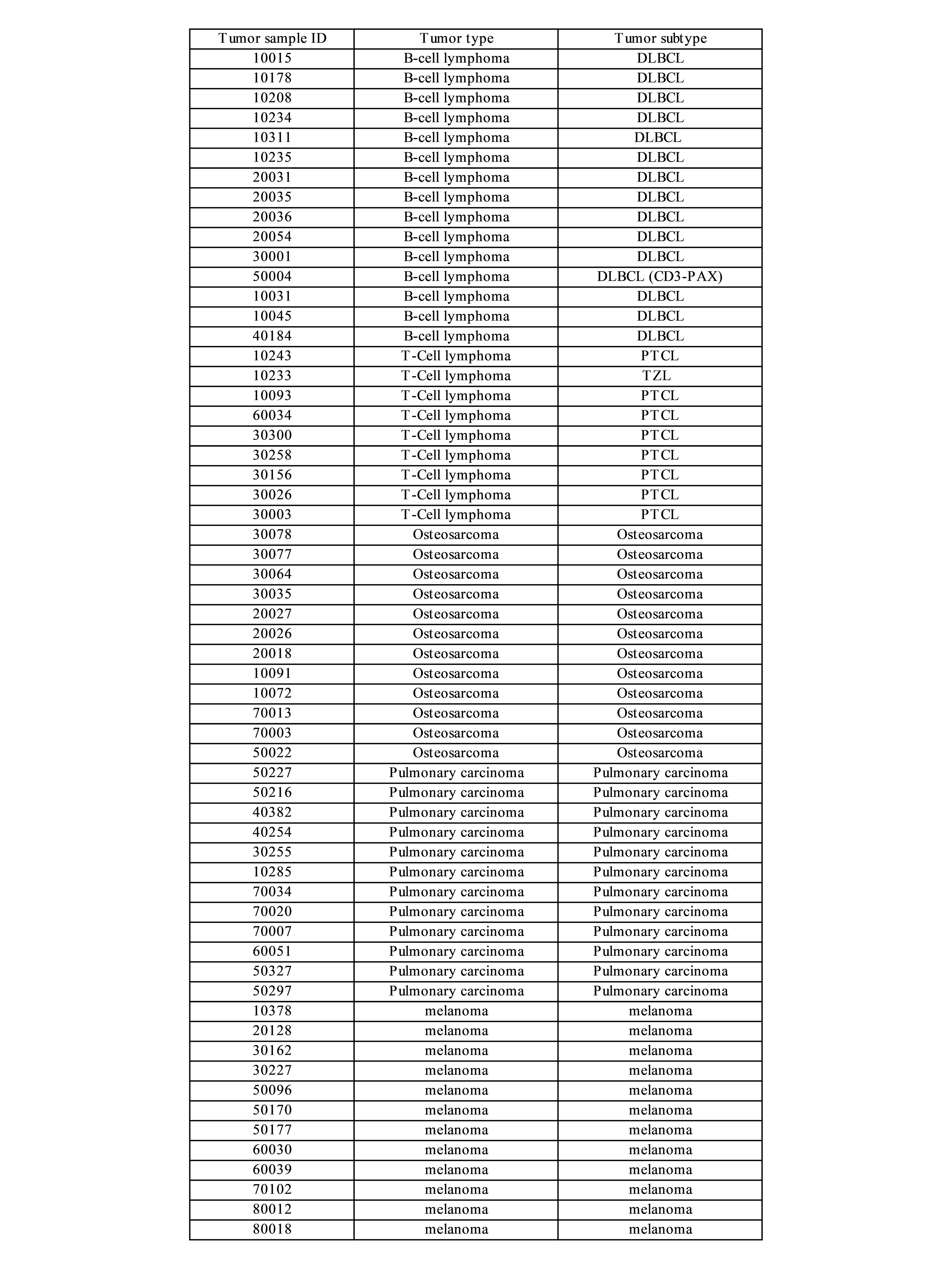

Supplement: S1 Fig — (TIF) [file pcbi.1009450.s001.tif]

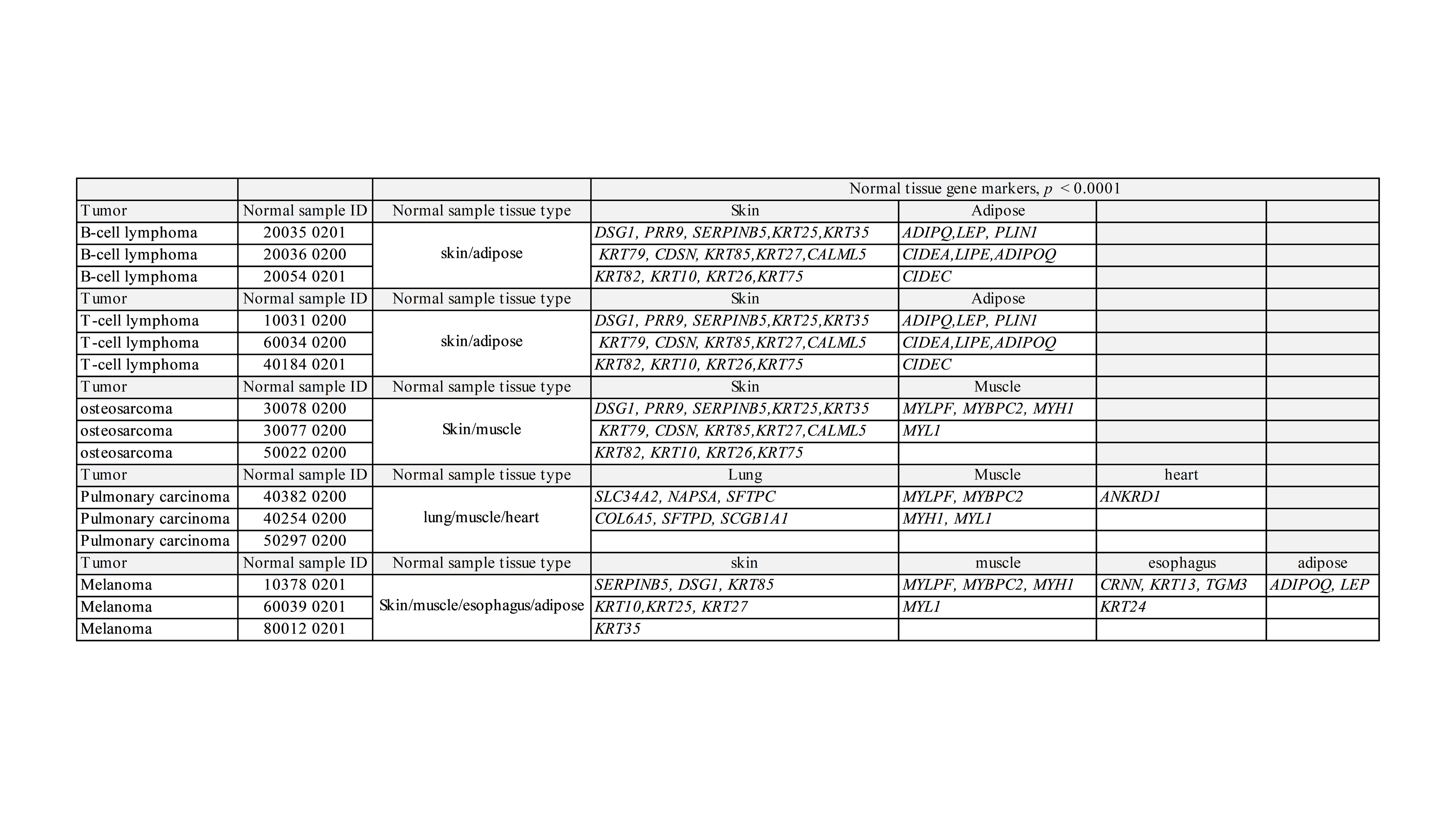

Supplement: S2 Fig — Normal samples were from the same dog as the tumor samples and were taken as near the tumor site as possible but far enough away so as not to include any neoplastic tissue. The top 200 expressed genes from each normal sample were submitted to the Tissue Specific Expression Analysis Tool (TSEA) [44] to identify the origins of the normal samples. (TIF) [file pcbi.1009450.s002.tif]

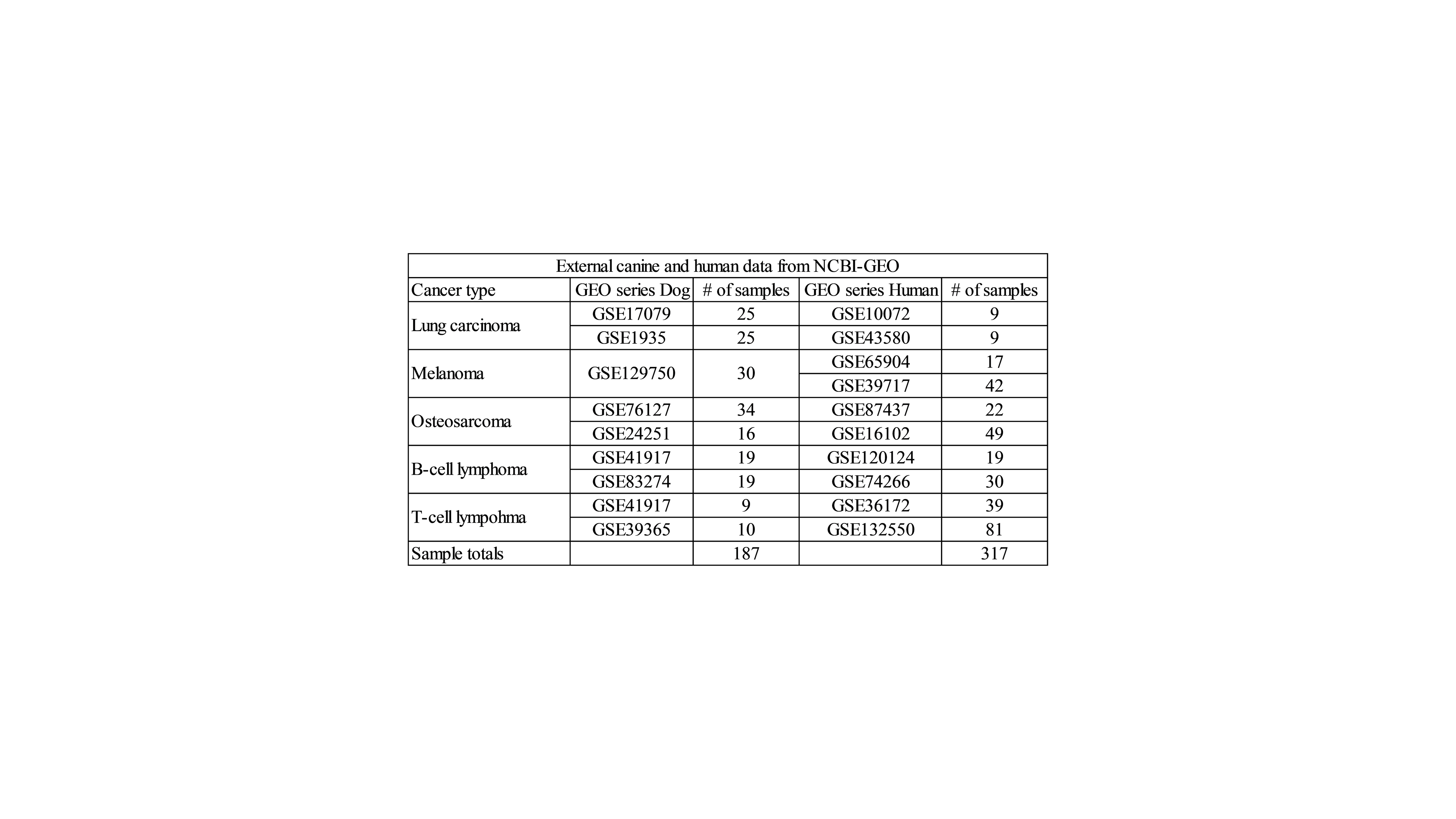

Supplement: S3 Fig — (TIF) [file pcbi.1009450.s003.tif]

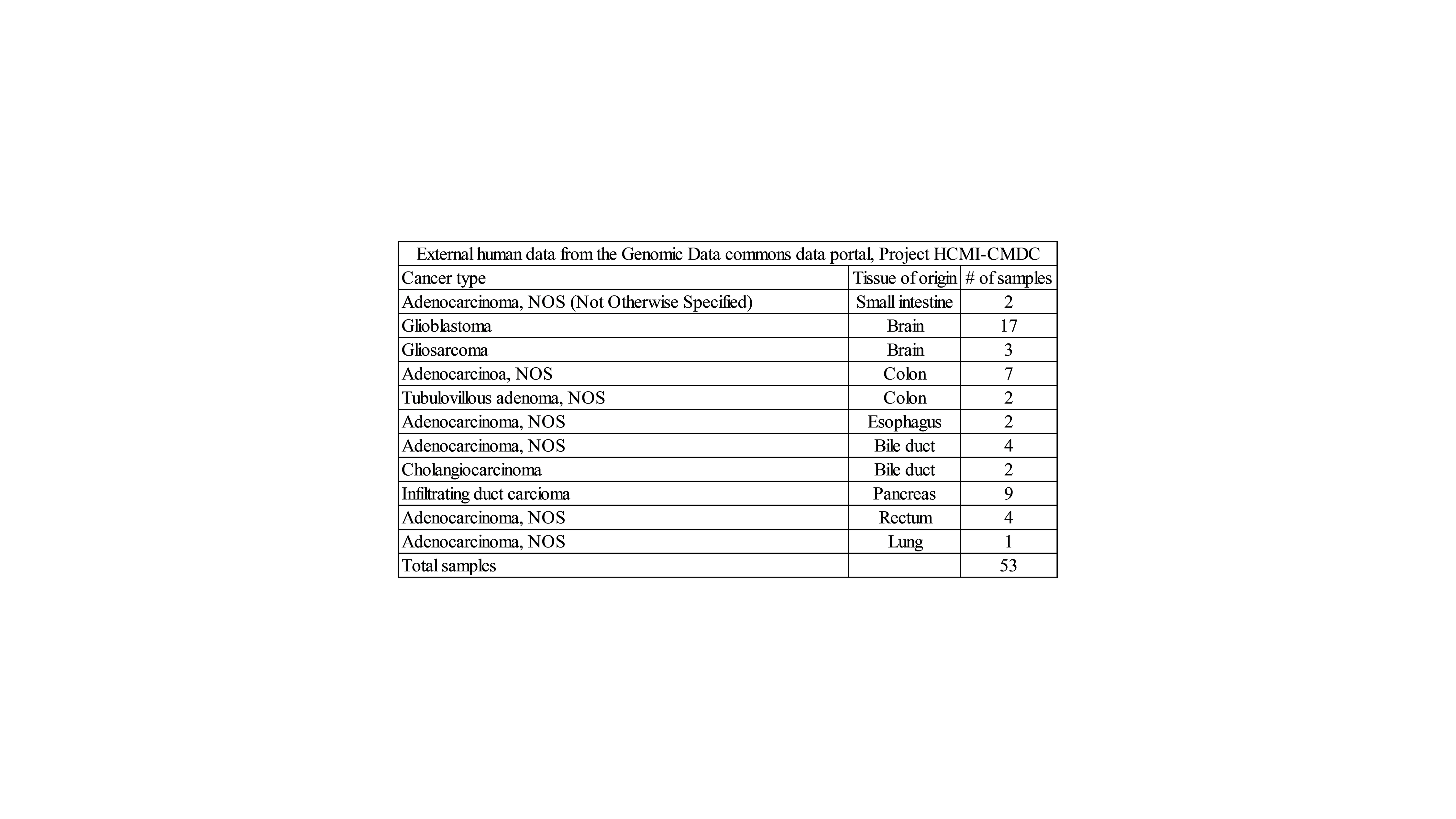

Supplement: S4 Fig — (TIF) [file pcbi.1009450.s004.tif]
