# Supplementary material for: Transcriptomic profiling in canines and humans reveals cancer specific gene modules and biological mechanisms common to both species
Source: PLoS Comput Biol. 2021 Sep 27;17(9):e1009450. doi: 10.1371/journal.pcbi.1009450 (PMC8523068; doi:10.1371/journal.pcbi.1009450)
Supplement: S8 File — (DOCX) [file pcbi.1009450.s012.docx]

**Sample purity as represented by tumor/stroma ratio**

**S1_File.xlsx** contains 5 tabs one each for PULM, MEL, OSA, BLSA, and TLSA. In each of these tabs total tissue area, tumor tissue area, and stroma tissue areas are given in columns F, G, and H, respectively. Tumor/Stroma ratios are given in column J. We used the Tumor/Stroma ratios to color-code the scatter plot in **Fig** 3E of the main text. This result is shown in **Fig** S8-1 below.


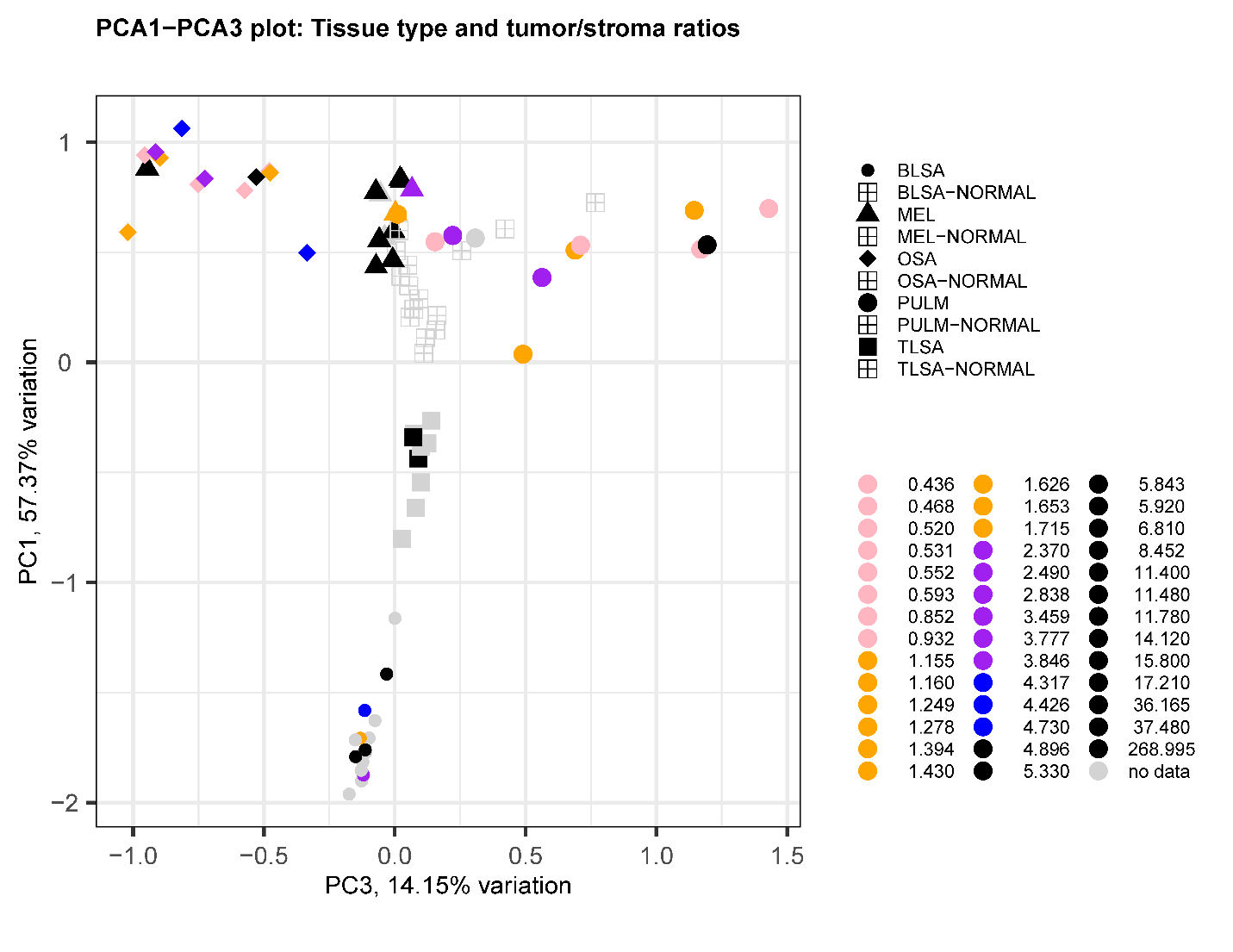


**Fig** S8-1. A biplot showing positions of the various tumor sample points in PCA space. The sample points are shaped according to tumor type (legend, upper right) and color coded according to their Tumor/stroma ratios (legend, lower right).

Analysis of the biplot shows that the sample points for each cancer occupy distinct and separate regions of PC1, PC3 space. It is not possible, within or between cancer types, to separate out the high tumor/stroma ratio samples from the low tumor/stroma ratio samples. Since all samples of a given cancer type occupy the same region of PC1, PC3 space, independent of the tumor/stroma ratio of the samples, we conclude that sample purity (as measured by tumor/stroma ratio) is not a confounding factor in these experiments. We define confounding factor as that which causes the spread in sample points for each cancer to become so large that samples of each cancer type can no longer be distinguished by their positions in PCA space.

One hypothesis that attempts to explain this is as follows: There is a lot of cross talk between tumor and stroma, so it is probably the case that expression of genes in the stroma is correlated with expression of genes in the tumor. The gene co-expression modules that we determine from the data are groups of genes with correlated expression patterns across the sample set. Since tumor and stroma genes are correlated due to crosstalk, it is likely that these co-expression modules contain genes from both tumor and stroma. For samples with high tumor/stroma ratios, the tumor genes contribute most to overall module expression. For samples with low tumor/stroma ratios it is the stroma genes that contribute most. In either case the module expression, which is an average over the individual tumor and stroma genes of the module remains relatively stable (more or less) and the unique module expression profile of each cancer retains this stability as well. The unique module expression profiles are responsible for the distinct and separate positions of the cancer samples in the biplot.

This hypothesis may be valid only to a point. In the current study, the median value of the tumor/stroma ratio is 3.5, with maximum of 269 and minimum of 0.3. In general, the samples contain roughly 3 times more tumor than stroma. It may very well be the case that if this ratio dips too low, the stability in the module averages will deteriorate, and the positions of the cancer samples in PC1, PC3 space will start expanding outward from their current positions. Eventually at very low purity (small tumor/stroma ratios) the sample distributions for each cancer will overlap with the sample distributions of the other cancers, resulting in a situation where the cancer samples are no longer distinct from one another. Indications that this may happen can be seen in **Fig** S8-2 below.

| Sample type | AREA (PC1-PC3) | tumor/stroma ratio | Total samples/# dog breeds |
| --- | --- | --- | --- |
| Normal | 0.034 |  | 1.250 |
| PULM | 0.263 | 1.844 | 1.110 |
| OSA | 0.068 | 2.414 | 1.110 |
| MEL | 0.035 | 11.894 | 1.290 |
| BLSA | 0.016 | 12.082 | 1.500 |
| TLSA | 0.000 | 137.419 | 1.000 |

**Fig** S8-2. Canine sample purity versus Area (PC1-PC3). Canine sample purity is represented by (i) average tumor stroma/ratio across the samples comprising a particular tumor type (column3) and (ii) total number of samples of a given cancer type/# dog breeds represented in the sample set.

The **Fig** S8-2 shows that as the sample purity, represented by tumor/stroma ratio, decreases, the sample areas mapped out by each cancer (as seen in Fig 1) increases quite dramatically. The sample purity, represented by (Total number of samples per cancer)/(# dog breeds represented in the sample set) remains relatively fixed near a value of 1. Considering the behavior of the PC1-PC3 areas mapped out by each cancer as a function of Tumor/stroma ratio it is not a stretch to think that for very low purity values, these areas mapped out by each cancer sample set begin to intermingle. In the current work, however, the tumor/stroma ratios are more than high enough to keep the different cancer samples in distinct and separate regions of PCA space, so this confounding factor situation has not yet manifested .

Computational notes:

- We only used data for which dog breed, tumor and stroma areas are known.
- For the samples associated with each cancer we remove outliers that had positions in PCA space less that Q1 – 1.5(IQR) or greater than Q3 + 1.5 IQR, where Q1 and Q3 are first and third quantiles along PC1 or PC3, and IQR is the interquartile range along PC1 or PC3.
- We assume that each cancer sample set maps out a convex hull polygon area. This area is computed using the “Spatial and Space-Time Point Pattern Analysis (splancs)” package in R ([https://CRAN.R-project.org/package=splancs](https://cran.r-project.org/package=splancs) ).
- The data used to run the above computations is contained in file **S8_File.xlsx**

**Sample purity as represented by breed heterogeneity**

The PCA areas mapped out by the samples of each cancer type are really produced by a combination of tumor/stroma and breed variations within the data sets associated with each cancer. To deconvolute these two parameters we overlayed dog breed information onto the data shown in **Fig** S8-1 to obtain **Fig** S8-3 below:


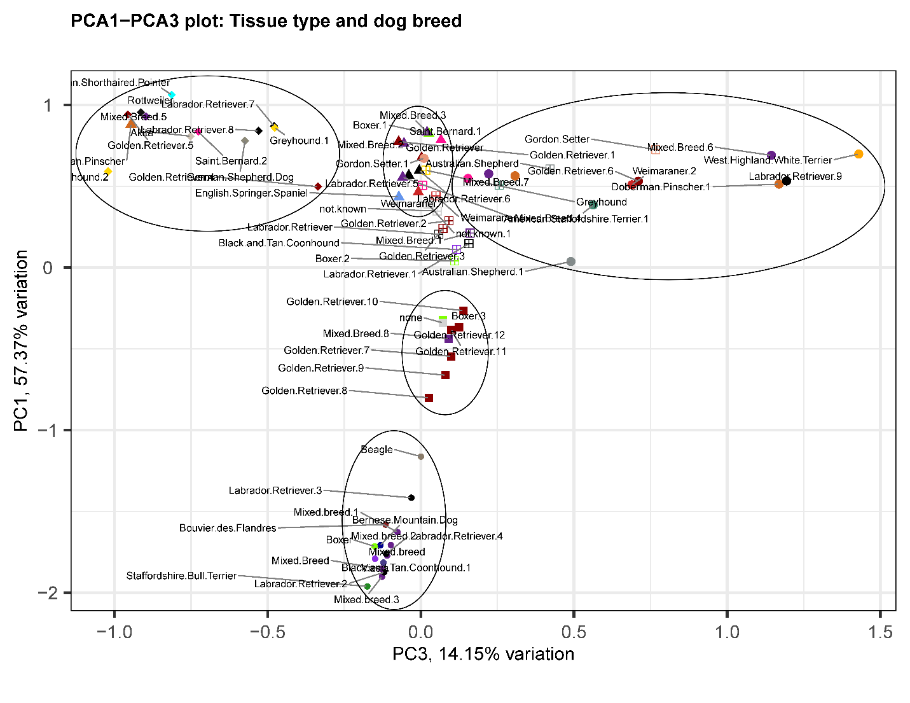


**Fig** S8-3. Dog breed information mapped to the PC1-PC3 biplot of **Fig** S8-1.

Expanding each of the tumor local areas of **Fig** S8-3 we obtain **Fig** S8-4 below which contains 5 expanded plots, one for each tumor.


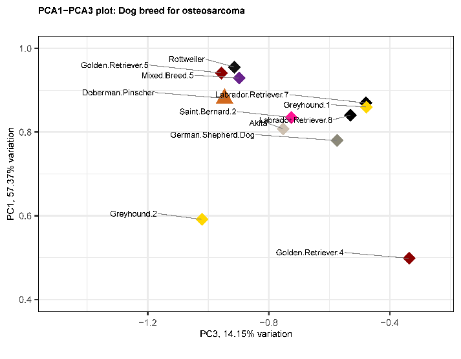

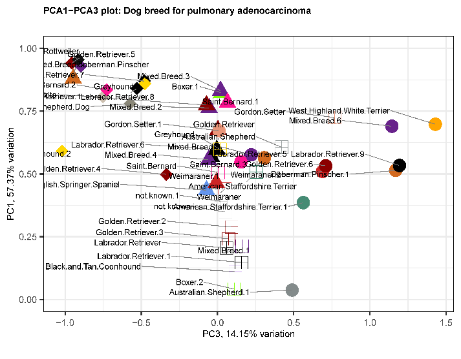


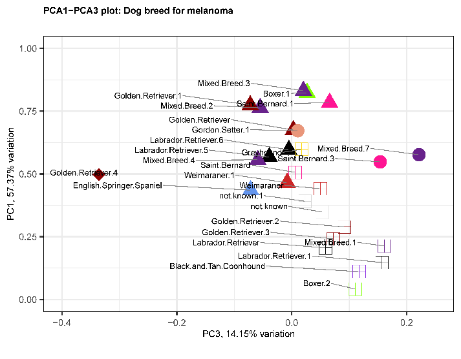

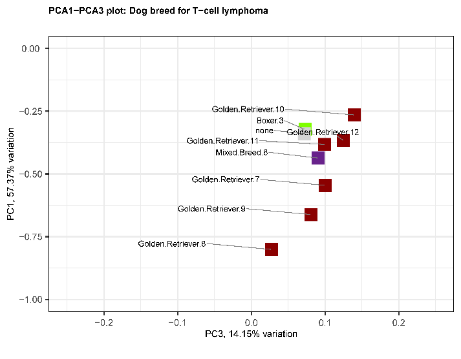

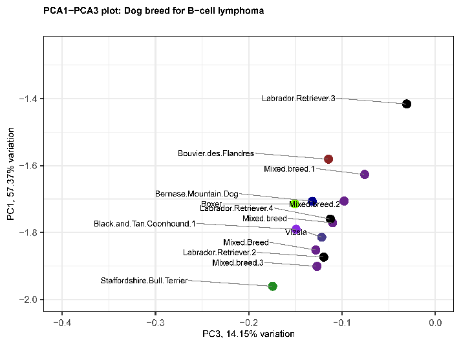


**Fig** S8-4. Expanded views of sample data associated with each of the 5 cancer types.

A study of the details of each tumor area shows that the breeds are not stratified within tumor type. There simply are not enough examples of each breed within the sample sets for each cancer to observe it (if such stratification exists). As such, aggregating samples by breed before co-expression analysis not warranted.

We can, however, surmise on the variance due to breed. To get a good idea of the data spread due to breed we need to go to a case where the tumor/ stroma ratio is fixed, while dog breed varies. We find that situation in the normal data for MEL, OSA, BLSA, and TLSA which all come from normal tissues taken from skin with a juxtaposition of adipose tissue. The idea of tumor/stroma does not exist here so presumably the data spread is completely dependent on dog breed (all else being equal). **Fig** S8-2, row 1, shows the normal sample area = 0.034 at a species purity value of 1.25. This species purity is very close to that of the Melanoma tumor samples, which have a sample area = 0.035 and a tumor/stroma ratio of 11.894. For melanoma each sample, on average, is 92% tumor and 8% stroma. Therefore, we can assume that variations due to tumor/stroma differences are small, and that the species diversity (7 out of 9 melanoma samples come from different dogs), contributes almost entirely to the 0.035 melanoma surface area on the PCA biplot of **Fig** S8-2.We would say then that variation due to tumor/stroma impurities = 0.01, variation due to species impurities = 0.034, the sum of which make 0.035, for Melanoma.

To deconvolute the rest of the data in this way, we would have to run RNA-seq on normal data associated with all of species purity values in **Fig** S8-2.

These are rough approximations, at best, however the cancer samples, and normal samples separate well in PC1-PC3 space, nonetheless. So, issues associated with impurities (species or tumor/stroma) are not large enough to confound the results.
