# Supplementary material for: Transcriptomic profiling in canines and humans reveals cancer specific gene modules and biological mechanisms common to both species
Source: PLoS Comput Biol. 2021 Sep 27;17(9):e1009450. doi: 10.1371/journal.pcbi.1009450 (PMC8523068; doi:10.1371/journal.pcbi.1009450)
Supplement: Attachment — Submitted filename: GT_response_to_reviewers_letter_PCOMPBIOL-D-21-00257.docx [file pcbi.1009450.s014.docx]

Dear Dr. Gallo,

Please see below for original text that you sent containing instructions for resubmitting a revised manuscript and the reviewer responses to the manuscript. Our responses to the reviewer’s comments are shown in red and cyan below and the revised manuscript (attached with this letter) has been color coded appropriately to reflect changes suggested by the reviewers. Also attached is a clean version of the manuscript).

If there is anything else that you need, please let me know.

Sincerely

Gregory J. Tawa

**___________________________________________________**

**Gregory J. Tawa**

**Modeling and Informatics Lead**

**NIH NCATS (National Center for Advancing Translational Sciences)**

**Therapeutic Development Branch**

**DIVISION OF PRE-CLINICAL INNOVATION (HN92)**

**9800 MEDICAL CENTER DR-BLDG C**

**ROCKVILLE MD 20850**

| **Office phone: 301-827-7177**  **Mobile: 240-550-0749**  **Email:Gregory.tawa@nih.govv** |  |
| --- | --- |

Decision from PLOS Computational Biology (PCOMPBIOL-D-21-00257) - [EMID:6497e42bd7e365a8]

Dear Dr. Tawa,

Thank you very much for submitting your manuscript "Transcriptomic profiling in canines and humans reveals cancer specific gene modules and biological mechanisms common to both species" for consideration at PLOS Computational Biology.

As with all papers reviewed by the journal, your manuscript was reviewed by members of the editorial board and by several independent reviewers. Considering the reviews (below this email), we would like to invite the resubmission of a significantly-revised version that considers the reviewers' comments.

We cannot make any decision about publication until we have seen the revised manuscript and your response to the reviewers' comments. Your revised manuscript is also likely to be sent to reviewers for further evaluation.

When you are ready to resubmit, please upload the following:

[1] A letter containing a detailed list of your responses to the review comments and a description of the changes you have made in the manuscript. Please note while forming your response, if your article is accepted, you may have the opportunity to make the peer review history publicly available. The record will include editor decision letters (with reviews) and your responses to reviewer comments. If eligible, we will contact you to opt in or out.

[2] Two versions of the revised manuscript: one with either highlights or tracked changes denoting where the text has been changed; the other a clean version (uploaded as the manuscript file).

Important additional instructions are given below your reviewer comments.

Please prepare and submit your revised manuscript within 60 days. If you anticipate any delay, please let us know the expected resubmission date by replying to this email. Please note that revised manuscripts received after the 60-day due date may require evaluation and peer review like newly submitted manuscripts.

Thank you again for your submission. We hope that our editorial process has been constructive so far, and we welcome your feedback at any time. Please don't hesitate to contact us if you have any questions or comments.

Sincerely,

James Gallo
Associate Editor
PLOS Computational Biology

Florian Markowetz
Deputy Editor
PLOS Computational Biology

***********************

Reviewer's Responses to Questions

**Comments to the Authors:
Please note here if the review is uploaded as an attachment.**

Reviewer #1: This is a straight forward paper and I have no issue with the methodology or the conclusions. However, it is purely observational with no functional data or analysis beyond description. If the editor and journal are fine with this, then publication is appropriate.

Reviewer #2: The authors describe a gene expression study based on RNA sequencing of 225 tumor and normal samples from 12 tumor samples and 3 matched controls for each of five canine cancer types: pulmonary carcinoma, melanoma, osteosarcoma, and B- and T-cell lymphoma from 60 dogs. They use a unique unsupervised clustering method to identify five co-expression modules and cancer type-associated expression profiles that also support shared cancer
biology between canines and humans for these cancer types. This is an important and well-designed study in the field of canine and comparative transcriptomics not only for what it reveals of transcriptomic similarities between five canine and human cancer types, but also due to the framework through which it rigorously establishes those similarities. Mostly minor needs are highlighted for revision to improve clarity and understanding of the value of the approach the authors have taken in the context of standard approaches and existing knowledge.

1. Minor comments:(Note: The text of the manuscript has been marked in red associating them with responses to Reviewer 2’s comments)
a. Although this study is focused squarely on transcriptomics and expression, it would nonetheless be helpful to add some perspective in the Intro and Discussion on the genomic (mutation-based) underpinnings at play in canine and comparative cancer genomics studies and that hold relevance for downstream transcriptional differences and/or possible tumor subtypes. This is particularly true since the genomic underpinnings of some of these tumor types are increasingly well-understood. Are there any possible connections, for example, between SETD2 mutations in osteosarcoma and any of the genes or expression modules discovered here? Any reason that possible similarities or differences exist between canine and human linked back to genetic underpinnings?

The reviewer makes an excellent point here. As such we have added material to the introduction (4’th paragraph) to give some perspective on mutations, cancer, and comparative oncology . We also added a sentence at the end of the 6’th paragraph of the introduction stating that the gene annotations can be used to link expressed genes to mutation events. In the discussion we then try as much as possible to form such linkages between our cancer genes and mutational events that drive the cancers. See additional discussion for Osteosarcoma (bottom of third paragraph, page 9), B-cell lymphoma (second paragraph page 10), Melanoma (third paragraph page 11), and Pulmonary carcinoma (second paragraph, page12).

b. Is there no value at all in performing more traditional gene expression analysis such as assessment of individually significantly differentially expressed genes in each tumor type, exploration of clusters based clinical features, etc.? It seems like it would be particularly effective to do that here since you have such a great set of replicates and sampling of tumor types. This could even be described preceding the unique approach here taken to highlighting co-expression modules and their constituents. Then, the relative value of each approach could be commented on.

In response to the reviewers comment we ran a conventional differential expression analysis on individual genes within each module to show the *p* values for comparison of the gene activation for the cancer samples versus normal samples. Tables 2-6 and supplementary file S7 have been modified to reflect this fact. This data is given in the columns titled: “Average activation across normal samples”, “average activation across cancer (PULM, MEL, OSA, BLSA, TLSA) samples”, “BH corrected *p*”.

We have clustered the genes into gene modules and associated them with cancer type, however, we do not have additional clinical data to associate gene clusters to clinical outcomes.

The reviewer suggests describing the traditional approach and comparing it to our unique approach. In response to this we have added a large paragraph in the discussion at the bottom of page 13 and top of page 14 that addresses this point.

c. Pg. 3 - How were the samples divided into three replicates? Was this at the tissue level, analyte level, sequencing library level, etc.? Methods makes it sound like RNA was extracted from a subdivided piece of tumor, but it’s not totally clear.

Please see methods section, subsection titled “RNA extraction and Quality Control”. Within that section the wording has been changed to:

“One tissue biopsy for each sample was used and from it we extracted 2 μg of RNA using an RNeasy Fibrous Tissue Mini Kit (cat No./ID:7404) (*58*).

Then in the subsection titled “RNA-Seq Experiments” we modified the first sentence as follows:

“Each RNA sample (75 altogether) from the single tumor biopsy was divided to make 3 libraries for RNA-Seq.”

Also see paragraph #1 of the results, top of page 4, where the above wording was also used.

To answer the reviewer’s question regarding at what point the replicates were made: The replicates were made at the sequencing library level, this should be clear to the reader now.

d. Like “d,” was tumor content somehow verified in each section? How do you know that you were sequencing primarily tumor and not normal stroma and how might you account for variability in tumor content between the replicates if they are from subdivided tumor sections?

The tumor content was verified. This information is given in supplementary file **S1** titled “S1-Biospecimens.xlsx. In it you will see that for most samples the following information is given:

Total Tissue Area

Tumor Tissue Area

Stroma Tissue Area

So, our samples contain tumor and stroma . On average, the samples are 80 % tumor and 20% stroma. Again, see supplementary file S1. This wording was placed in paragraph 1 of the Results.

Note that the mix of tumor and stroma cells is typical for patient samples as they are never 100% tumor. It is an advantage for a bulk genomics project, that dogs recapitulate that landscape in humans, unlike mice where the tumor does not necessarily co-evolve with relevant stroma.

The samples are NOT from subdivided tumor sections, they are from a single biopsy.

e. Pg. 3 - The first section of the Results doesn’t really seem to be an independent result of particularly advanced analysis worthy of its own segment, but perhaps should be a comment in the following section when addressing various elements of the cohort composition.

At the reviewer’s suggestion, we removed this section and absorbed the material at the back end of the results subsection titled “Biomarker hypotheses selected from the primary modules of each cancer bear significant evidence linking them to their associated cancer.” It seems more appropriate to add this material at the end of the results stating that the results are likely to be widely applicable because our sample set represented a typical cross section of dog breeds owned in the USA. See bottom of page 7, top of page 8 in the text.

f. Pg. 3 – Recommend retitling the second Results section as “Five gene co-expression modules represent coordinated activation patterns of gene aggregates associated with the 5 types of cancer” or something similarly more declarative rather than the current title that’s somewhat equivocal.

This has been modified according to all reviewer’s suggestions.

g. Pg. 3 - It’s not immediately clear how we went from a 24,580 x 225 sample matrix to a 4,115 x 75 sample matrix.

See the wording in this section (now first paragraph of page 4). It reads:

“After initial processing of the resulting sequence reads, we arrived at a 24,580 gene by 225 sample raw count matrix. See supplementary file **S3**. Further processing the count matrix by (i) removing canine genes with low read counts, (ii) removing canine genes with low variance, (iii) removing canine genes with no associated human homologs, and (iv) aggregating replicates, we arrived at an expression matrix composed of 4115 genes (rows) and 75 samples (columns). See methods sub-section titled “RNA-Seq data processing” for further details.”

For example, regarding the variance, we calculated it for each gene across the sample set. We sort the genes decreasing by variance, and we get rid of genes in the lower half of the list. This immediately cuts the gene set down to 12240. This is discussed in the methods section and is based on work in reference (65). This references discusses how gene filtering (in the way that we have implemented) increases detection power for high throughput screening experiments.

h. I noticed a handful of run-on sentences such as the following on page 3: “We determined the expression profile of each of these modules across the samples, these are shown in Fig. 2.”

We replaced this with (first paragraph now page 4) with:

**“Fig**. **2** shows the expression profiles of each of these modules across the samples**.”**

We found other such instances of run-on sentences in the text and replaced them as so done above.

i. There’s a lot of unusual nomenclature in here that can be difficult to relate back to the underlying biology and/or is perhaps even not specific enough, so it quickly gets confusing (at least for me). Can any of this be simplified or reworded or more precisely defined? For example, “coordinated activation patterns,” “gene aggregates,” “coupled transcriptional events associated with multiple overlapping and intertwined cancer mechanisms,” “uniqueness property,” and even “co-expression module” are not immediately well-defined or intuitively understandable.

The terms coordinated activation patterns and gene aggregates have been removed from the text and replaced with simpler wording. For example, on page 4 end of paragraph 1 we replaced:

“The profiles exhibit coordinated activation patterns of different gene aggregates associated with each of the 5 cancers. “

With

“The expression patterns of the modules are distinct, typically exhibiting significant activation in one or two cancer types.”

Another example on page 2, paragraph 3, we replaced:

“The aggregate properties of a group of genes, (*22, 23*) however, may be easier to detect. If such aggregate properties are considered, data complexity is reduced, allowing elucidation of cancer similarities and differences within and between canine and human species.”

With

“The average expression of a group of genes, (22,23) however, may be easier to detect. If such expression averages are considered, data complexity is reduced, allowing elucidation of cancer similarities and differences within and between canine and human species.”

Similar replacements have been made throughout the text.

The sentence (Now first paragraph of Page 4):

“The Z matrix contains gene activation patterns of the 5 cancer types and represents the coupled transcriptional events associated with multiple overlapping and intertwined cancer mechanisms.”

Has be replaced with:

“The Z matrix contains gene activation patterns of the 5 cancer types. Each pattern represents the transcriptional events associated with the biological mechanisms at play for each cancer.”

The term “uniqueness property” has been removed from the text and replaced with wording easier to understand. For example, on page 5 (formerly page 4) first sentence of the second paragraph we replaced:

“Because of the uniqueness property of the module activation profiles, we reasoned that these profiles could be used to build cancer classification models.”

With

“Because each cancer exhibits significant activation in a different module, we reasoned that the gene modules could be used to build accurate cancer classification models.”

With regard to the term “co-expression module” we added a precise definition of co-expression module to the second to last paragraph of the introduction:

“A co-expression module corresponds to a group of genes that have a similar expression pattern across samples analyzed. Genes in a co-expression module are of interest because such genes are thought to members of the same biological pathways or part of the same transcriptional regulatory program.”

With this definition in mind from the beginning, the reader should not be confused later when the term “gene co-expression module” is used.

Reviewer #3: This manuscript provides novel insights into possible shared transcriptional pathways between human and canine malignancies as well as highlighting the potential utility of using spontaneous canine malignancies as a model for human cancers. The authors use RNAseq data from 5 different cancer types to find genes which form co-expression modules and later apply these modules to human cancers to show cross-species utility of such findings. (Note: The text of the manuscript has been marked in cyan associating them with responses to reviewer 3’s comments)

Main Text:
It is unclear where the “matched controls” come from, what type of tissue is used, and if it is from the same animal (may be in supplementary tables we don’t have access to?). This is of specific importance as it determines whether the proposed biomarkers are relevant in determining cancerous vs non-cancerous tissue or, alternatively, simply identifying what type of tissue has been collected.

We augmented the table caption of supplementary table S1. It now reads:

“Table S1. Canine sample histologies. Normal samples were from the same dog as the tumor samples and were typically taken as near the tumor site as possible but far enough away so as not to include any neoplastic tissue.”

Comparing each module activation against the average activation across all normal tissue types of all origins is possibly just selecting for tissue-specific transcription rather than cancer-related gene expression.

We changed this according to the reviewer’s suggestion. Now we compare the module activation of each cancer to only the matched normal sample associated with each cancer. This activation is an average activation across only normal tissue samples matched to the cancer type in question. All reference to pooled normal samples has been removed from the paper. Figure 3 has been modified accordingly.

Analysis of module activity should include classification of normal tissue samples as well as it relates to tissue type as this is critical in presenting this data as cancer-specific biomarkers.

“We studied this problem in detail. The normal samples come from the CCOGC, with the information that the normal should be collected “near” the tumor site. We modified the table S1 caption to reflect this fact. But no further detail is given from the CCOGC as to the precise location from which the normal samples were harvested. To try to nail down the identity of these normal samples we began by running a PCA analysis of the tumor samples and matched normal samples where each sample is represented by a set of 5 module activations, each activation compiled as an average across all genes of the module for each sample representing the tumor and normal. We added this PCA plot to Figure 3 to show how the normal samples are juxtaposed relative to the tumor samples. The PCA plot shows excellent separation of the tumor samples (small circles) which bodes well for classification models based on modules. The normal samples, however, are a different story. For BLSA, TLSA, MEL, and OSA, the normal samples are all very similar, which we can tell by their location near the center of the PCA plot. They are also different than the respective tumor samples that they are associated with. The tumor samples appear roughly at the edges of the plot. This implies that the normal samples were collected from a similar tissue source but at a site different than the tumors. The PULM normal samples, however, occupy the same region in the PCA plot as the PULM tumor samples. This implies that the PULM normal samples were collected from healthy lung tissue, in the vicinity of the tumor.”

---------------------------------------------------------------------------------------------------------------------

“To validate these observations, we compiled a list of the top 200 expressing (read counts) genes for each normal sample, and then performed a tissue specific enrichment analysis using the Dougherty Web-based TSEA tool (Dougherty, Nucleic Acids Res, 2010, 28(13) 4218, http://genetics.wustl.edu/jdlab/tsea/). The tissue marker genes pulled for the various normal samples, without a doubt identified the B and T cell lymphoma normal tissues as skin with an admixture of adipose tissue , the osteosarcoma and melanoma normal samples were mainly skin with an admixture of muscle tissue, the pulmonary carcinoma normal samples were mainly lung with an admixture of muscle tissue. These results are consistent with the positions of the normal samples on the PCA plot.

The result of these observations means that for PULM, we may be able to deconvolute tissue specific transcription from cancer specific transcription based on the p values for comparison of tumor to normal gene expression. For the other tumor types, this deconvolution, based on p values, is not possible because the normal samples come from a different tissues source than the tumor samples. We therefore must appeal to the gene annotations to decide which genes are related to the cancer and not just tissue specific translation.”

The discussion above the dotted line appears at the bottom of page 4, top of page 5 of the text to give more detail regarding the nature of the normal samples. Also, we added a PCA plot to Figure 3 (Figure 3E) to support the added text.

The discussion below the dotted line has been placed in the second paragraph of the discussion. We also modified Table S1 to include the results of the tissue specific enrichment analysis.

Figures:

**Figure 2:** This figure is difficult to interpret, and this data may benefit from being displayed as a heatmap with samples as columns and genes as rows to better appreciate the grouped activation. If this stays as a line graph, a key should be included for the genes each color represents.

In this case we decided to replace Fig. 2 with a heatmap as the reviewer suggests, with columns defined by samples and rows defined by genes. The gene activations are color coded from black (no activation) to white (high activation).

Figure 3: This data may be better presented as a boxplot or with individual data points showing so that the distribution of such values is better represented rather than the mean and standard deviation. This data should also be derived from appropriately labeled samples as mentioned below.

According to the reviewer’s suggestion we added box plots to the Figure (Figure 3D), and we removed the original bar plots. The box plots show that the distribution of individual data points is not normal (space between quartiles is not identical). This is not captured by quoting averages and standard deviations with the bar charts as we did previously. We added text supporting the box plot image on page 4, last paragraph. We also added a PCA plot to Figure 3. This plot shows the spatial relationships between the data points. The tumor samples (circles) are seen to be well separated. Not so for the normal samples, this was discussed above. Text supporting the PCA plot is located at bottom of page 4, top of page 5 as discussed above.

Figure 5: This table should include the number of genes within the model as well as the number of genes within the defined set. Tables 2-6 show that many of the “enriched” gene sets in each module only contain a single gene from the module, which limits the utility of using such gene set annotations to aid in defining the module.

We do not have a Figure 5, so we are not sure what the reviewer is referring to? We assume that it must be Table 1 that is being referred to. As such we modified Table 1 by adding the number of genes in each module below the module designation. For number of genes within the “defined set” we think that the reviewer means the number of genes associated with each annotation? For that we found it easier to add this information to Tables 2-6. To this end we modified Tables 2 -6 in the following fashion: Below the annotations we added two rows of numbers. The first row shows the number of genes in the module that are mapped to the annotation, the second row shows the number of highly expressed module genes (activation > 1.5) that are mapped to the annotation. We believe that these two new rows of data are “genes within the defined set” as the reviewer says above. This should clear up (somewhat) issues regarding single genes from modules associated with an annotation. Tables 2-6 include only genes with activation > 1.5 and annotations specific to the cancer being described. The full tables are given in supplementary information (Supplementary file 7). We added wording to bottom of page 19, top of page 20 in the Methods section that supports these additions to the tables.

It may be more useful to evaluate the same data sets taking into account the overall activation of each genes using GSEA (<https://www.gsea-msigdb.org/gsea/index.jsp>) as it seems a single, barely activated gene should constitute gene set enrichment (for example, Table 2 – PTGIS in oxidation-reduction process). Such analysis should also account for the fact that the number of each genes in the set is limited by looking only at the intersection of canine and human genes, greatly reducing the number of genes within each set.

See discussion above. Now that we have added the two additional rows of data (as discussed above) one can look at PTGIS and see that it is one of 16 genes from module 1 that map to “oxidation-reduction process”. PTGIS is included in Table 1 because it has activation > 1.5 which was our defined cut-off for considering a gene biomarker candidate.

In addition, we ran a conventional differential expression (Wilcoxon cancer vs control) analysis to show the p values for comparison of the gene activation for the cancer versus matched normal conditions. For tables 2-6 and supplementary file S7 this data is given in the columns titled: “Average activation across normal samples”, “average activation across cancer (PULM, MEL, OSA, BLSA, TLSA) samples”, “BH corrected p”. With this additional data added to the tables, one can look at supplementary file S7, tab titled “Module 1 gene detail for PULM” (expanded versions of Tables 2-6) and see that PTGIS and many of the 15 other genes that have as annotation “oxidation-reduction process” are not significantly activated relative to the normal sample. Hence “oxidation-reduction process” is really associated with tissue specific transcription, and not anything to do with pulmonary carcinoma.

See discussion at the bottom of page 11 and top of page 12 that discusses tissue specific versus cancer specific expression with regards to pulmonary carcinoma. This discussion is possible now that we have added the differential expression analysis as the reviewer suggests.

Supplementary Data: Major reservations for this manuscript come largely from the information revealed in the supplementary data detailing the misclassification of T-cell lymphoma samples and the decision to present the entirety of the main manuscript with these samples included in the incorrect group. The authors suggest that “the module activation profiles are essentially unchanged” though activation of module 4 in the TLSA samples nearly halves upon proper classification while module 5 activation within the same group increases by roughly 25% in the same group. Reclassifying the samples greatly increases the specificity of module 5 for TSLA. These samples also make up about 25% of the total samples used in this analysis, so it is disingenuous to conclude that the rest of the manuscript should not be altered to reflect the appropriate classification of such samples.

We thank the reviewer for comments regarding the misclassification discussion in the supplementary material. To remedy this problem, we started from the beginning with the proper classification of samples. Supplementary table S1 has been modified so that the three mis-classified T cell lymphoma samples are now B-cell lymphoma samples, resulting in 15 instead of the original 12 B-cell samples, and 9 instead of the original 12 T cell samples. Starting with this correct classification, we re-ran the calculations from the beginning. The results of the recalculations are shown in the Figures and Tables of the main text. All discussion of “misclassification” has now been removed from the supplementary material and does not appear anywhere else in the text. In the heatmaps of Fig 2, we certainly see that module 5 activation is more specific to T cell lymphoma than it was before, and that module 4 activation is likewise diminished. This is also represented in the module activation values shown in Figure 3A.We believe that the manuscript is now far easier to understand and more honest, thanks to the reviewer’s comments.

**Have all data underlying the figures and results presented in the manuscript been provided?**
Large-scale datasets should be made available via a public repository as described in the *PLOS Computational Biology* [data availability policy](http://journals.plos.org/ploscompbiol/s/data-availability), and numerical data that underlies graphs or summary statistics should be provided in spreadsheet form as supporting information.

Reviewer #1: Yes

Reviewer #2: Yes

Reviewer #3: **No:** CanineCommons website was down at the time of access.

PLOS authors have the option to publish the peer review history of their article ([what does this mean?](https://journals.plos.org/ploscompbiol/s/editorial-and-peer-review-process#loc-peer-review-history)). If published, this will include your full peer review and any attached files.


If you choose “no”, your identity will remain anonymous but your review may still be made public.


**Do you want your identity to be public for this peer review?** For information about this choice, including consent withdrawal, please see our [Privacy Policy](https://www.plos.org/privacy-policy).

Reviewer #1: No

Reviewer #2: No

Reviewer #3: No

Figure Files:

While revising your submission, please upload your figure files to the Preflight Analysis and Conversion Engine (PACE) digital diagnostic tool, [https://pacev2.apexcovantage.com](https://pacev2.apexcovantage.com/). PACE helps ensure that figures meet PLOS requirements. To use PACE, you must first register as a user. Then, login and navigate to the UPLOAD tab, where you will find detailed instructions on how to use the tool. If you encounter any issues or have any questions when using PACE, please email us at [figures@plos.org](mailto:figures@plos.org).

Data Requirements:

Please note that, as a condition of publication, PLOS' data policy requires that you make available all data used to draw the conclusions outlined in your manuscript. Data must be deposited in an appropriate repository, included within the body of the manuscript, or uploaded as supporting information. This includes all numerical values that were used to generate graphs, histograms etc.. For an example in PLOS Biology see here: <http://www.plosbiology.org/article/info%3Adoi%2F10.1371%2Fjournal.pbio.1001908#s5>.

Reproducibility:

To enhance the reproducibility of your results, we recommend that you deposit your laboratory protocols in protocols.io, where a protocol can be assigned its own identifier (DOI) such that it can be cited independently in the future. Additionally, PLOS ONE offers an option to publish peer-reviewed clinical study protocols. Read more information on sharing protocols at <https://plos.org/protocols?utm_medium=editorial-email&utm_source=authorletters&utm_campaign=protocols>

*In compliance with data protection regulations, you may request that we remove your personal registration details at any time.* [*(Remove my information/details)*](https://www.editorialmanager.com/pcompbiol/login.asp?a=r)*. Please contact the publication office if you have any questions.*
