# Supplementary material for: Transcriptomic profiling in canines and humans reveals cancer specific gene modules and biological mechanisms common to both species
Source: PLoS Comput Biol. 2021 Sep 27;17(9):e1009450. doi: 10.1371/journal.pcbi.1009450 (PMC8523068; doi:10.1371/journal.pcbi.1009450)
Supplement: Attachment — Submitted filename: GT_response_v2_D-21-00257.docx [file pcbi.1009450.s015.docx]

Dear Dr. Gallo,

Please see below for original text that you sent containing instructions for resubmitting a revised manuscript and the reviewer responses to the manuscript. Our responses to the reviewer’s comments are shown in red, cyan, and tan below and the revised manuscript (attached with this letter) has been color coded appropriately to reflect changes suggested by the reviewers. Also attached is a clean version of the manuscript).

If there is anything else that you need, please let me know.

Sincerely

Gregory J. Tawa

**___________________________________________________**

**Gregory J. Tawa**

**Modeling and Informatics Lead**

**NIH NCATS (National Center for Advancing Translational Sciences)**

**Therapeutic Development Branch**

**DIVISION OF PRE-CLINICAL INNOVATION (HN92)**

**9800 MEDICAL CENTER DR-BLDG C**

**ROCKVILLE MD 20850**

| **Office phone: 301-827-7177**  **Mobile: 240-550-0749**  **Email:Gregory.tawa@nih.govv** |  |
| --- | --- |

Dear Dr. Tawa,

Thank you very much for submitting your manuscript "Transcriptomic profiling in canines and humans reveals cancer specific gene modules and biological mechanisms common to both species" for consideration at PLOS Computational Biology. As with all papers reviewed by the journal, your manuscript was reviewed by members of the editorial board and by several independent reviewers. The reviewers appreciated the attention to an important topic. Based on the reviews, we are likely to accept this manuscript for publication, providing that you modify the manuscript according to the review recommendations.

Please prepare and submit your revised manuscript within 30 days. If you anticipate any delay, please let us know the expected resubmission date by replying to this email.

When you are ready to resubmit, please upload the following:

[1] A letter containing a detailed list of your responses to all review comments, and a description of the changes you have made in the manuscript. Please note while forming your response, if your article is accepted, you may have the opportunity to make the peer review history publicly available. The record will include editor decision letters (with reviews) and your responses to reviewer comments. If eligible, we will contact you to opt in or out

[2] Two versions of the revised manuscript: one with either highlights or tracked changes denoting where the text has been changed; the other a clean version (uploaded as the manuscript file).

Important additional instructions are given below your reviewer comments.

Thank you again for your submission to our journal. We hope that our editorial process has been constructive so far, and we welcome your feedback at any time. Please don't hesitate to contact us if you have any questions or comments.

Sincerely,

James Gallo

Associate Editor

PLOS Computational Biology

Florian Markowetz

Deputy Editor

PLOS Computational Biology

***********************

A link appears below if there are any accompanying review attachments. If you believe any reviews to be missing, please contact [ploscompbiol@plos.org](mailto:ploscompbiol@plos.org) immediately:

Reviewer's Responses to Questions

**Comments to the Authors:
Please note here if the review is uploaded as an attachment.**

Reviewer #2: The authors have done an extraordinary job of addressing all of the reviewer comments.

**Thank you for your comments**

Reviewer #4: Tawa et al. have analyzed canine transcriptomics data in the context of existing human data to evaluate the comparative relevance of canine to human cancer. They show that tumour biology and biomarker candidates discovered in canines may translate to humans. Overall, the methods and results of this paper are well done and straightforward to follow.

Furthermore, the Authors have taken all of the reviewer's comments seriously and addressed them appropriately. I have no further comments or possible correction on the current manuscripts

**Thank you for your comments**

**Reviewer #5**: The authors developed an protocol that analyzes canine transcriptomic data in the context of existing human data to evaluate comparative relevance of canine to human cancer. They characterized five canine cancers, each exhibiting a unique module expression profile. The canine-derived models they generated successfully classified human tumors representing the same cancers. They also identified 104 biomarkers as diagnostic biomarker candidates. I only have some minor suggestions.

Figure 2. what is the unit for the y-axis?

The Figure 2 caption was modified, it now reads:

**Fig 2. Activation patterns of gene modules 1-5 across the cancer samples used in this**

**study.** Rows represent individual module genes, columns represent samples, color coding

represents activation ranging from low (green) to high 20 (red). Individual gene activations are Z-scaled read counts computed using Eq. (2).

To support this extra description in the caption we modified Fig 2 on the left hand side of each depicted module as follows (module 1 given as example)

which clearly defines the vertical axis by including gene designations g1, g2, …g32. Fig 2 at right center was modified as follows:

which associates the expression scale with A_g,s_ which is computed using Eq. (2) (see methods section of manuscript). Note that we change the color scheme from green to read at the request of one of the other reviewers

Figure 3. It seems that most of the values in Figure 3C are significant, whereas only a few are labeled with * in D. What are the criteria for being regarded as significant?
Tables. Please explain what 0 and 1 refer to and what the red color denotes.

We expanded the Figure 3 caption to cover the questions stated above. The caption now reads:

**“Fig 3. Comparison of module activations between cancer and normal samples.**

(A) Module activations for each of the 5 cancers studied. (B) Module activations of matched normal controls. (C) Module activation *p* values for comparison of cancer sample activation with the normal sample activation. Colored entries in (A)-(C) mark the values for the primary modules of each cancer type. Primary modules are defined as the highest expressed module with *p* < 0.05 for each cancer type. The primary modules are: module 1, cyan, for PULM, module 2, orange, for MEL, module 3, purple, for OSA, module 4, brown, for BLSA, and module 5, green, for TLSA. (D) Box plots showing distributions of primary module gene activations for each cancer and matched normal (Eq. [2]). Open circles are oulier genes. Red diamonds locate positions of the average activation across all genes within each module (Eq. [3]). The center of each notch is the position of the median activation. (E) PCA plot showing positions of cancer samples (small circles) and associated matched normals (large squares). Each sample is represented by a set of 5 module activations, each activation compiled as an average across all genes of each module.”

Yes, most of the module activations for each cancer in part C are significant because they are compiled as an average expression across 12 samples for each cancer type and ~ 100 genes for each module. That is a distribution of ~1200 gene expression values for each module activation so the statistical power of any comparison of module activation for cancer versus normal will be very high.

In Figure 3D there are no stars marking significance. What the reviewer may be thinking is that the red diamonds are marking significance. That is not the case, the red diamonds mark the location of the average activation value of module genes for each cancer and its matched normal. This is stated in the Figure caption. The criteria that we use to assign modules to each cancer, a.k.a. primary modules, is that we identify the module with the highest activation, that has *p* value < 0.05 for each cancer. This is where the color coding comes into play in Figures A-D.

Regarding the 0 and 1 and red coloring, we assume that the reviewer is talking about Fig 5. To address this, we augmented the Figure 5 caption, it now reads:

“Fig 5. KEGG and GO annotations for modules and associated cancers. Annotation matrix with rows enumerated by the different annotations and columns enumerated by cancer types and associated primary modules. Matrix elements are populated with 1 (and colored red) or 0 depending on whether the annotation is or is not associated with the module. The red color is included to highlight the block diagonal nature of the matrix. The column titled “# Modules” tallies the total number of modules that the annotations are associated with. The average BH corrected *p* values for this association is given in the last column of the Table. Only p values < 0.005 are shown. P values are shaded from red to white, with the highest *p* values in red transitioning to the lowest p values in white. See supplementary file S6-annotations_full.xlsx for the full set of annotations (*p* < 0.05).”

This clearly explains what 0 and 1 are, and why the red color is in the table.

We have also modified the Figure captions for **Figs** 6-10 to explain the 0 and 1 values and the red coloring. For example, the Figure 6 caption now reads:

**“Fig 6. Primary module 1 genes for pulmonary carcinoma.** Genes (activation > 1.5) and annotations shown are specific to pulmonary carcinoma in this study. In the table, red boxed 1 values indicated that the gene at far left in column 1 is associated with the annotation at top. White boxed 0 values indicate that the gene (at left) is not associated with the annotation ( at top). Pink boxes under BH corrected *p* value indicate genes with statistically significant activation difference between tumor and normal samples. Genes colored yellow and maroon are proposed novel targets for drug discovery. See supplementary file **S7-annotations_gene_detail.xlsx** which contains all module 1 genes for pulmonary carcinoma.”

This now clearly explains the 0, 1 and the red color. Figure captions 7-10 have been modified in similar fashion.

**Reviewer #6:** In this paper, Tawa, et. al., describes a study using gene expression from a series of canine tumor and normal tissue samples to identify gene expression modules that can classify tumor types in the context of both canine and human disease. I agree with the original reviewer 1 that this is a strictly descriptive paper (no functional analysis performed) and would benefit from modifying the focus of the discussion less to how these results recapitulate aspects of results already seen in other systems and more to what new science this analysis enables. Nonetheless, the approach is straightforward and if the editor and journal are fine with descriptive work, then this is fine.

The authors largely responded to the comments of reviewers 2 and 3. Find some additional minor concerns that need to be addressed below:

As a general note, all of the Figures that were part of the reviewer packager were very low resolution and when they involved small text in a table is was often illegible. Please double check that you have the appropriate resolution settings when you generate your Figures for publication.

We remade all Figures with higher resolution. They should be much sharper than previous.

The incorporation of the discussion around the common mutational landscape in canines and humans is somewhat awkward. I would recommend moving it earlier in the introduction to provide further justification for why canines are a useful model system to understand human cancer and then lead into gene expression and stay there.

As per the reviewer suggestion we moved this material to the first paragraph of the introduction where it fits helps to underscore canines as useful model systems for humans.

Intro paragraph 3, sentence 1: clarify “these studies.” Are “these studies” the studies that compare human and canine cancer biology to validate canines as a relevant model system? Or are “these studies” studies of cancer biology and experimental therapeutics?

To make this clear we replaced:

“A crucial first step in conducting these studies is careful selection of biological samples according to experimental design.”

With

“Access to high-quality canine samples is critical to understanding the complexities of canine cancer and potential translation of canine findings to human cancer research.”

It should be obvious now that “these studies” are studies that compare human and canine cancer biology to validate canines as a relevant model system.

Be consistent in bolding Figure references in the main text. For example the first reference to Fig 1A is bolded, but then Fig 1B and on are not.

Fixed.

Based on your previous responses to the original Reviewer 2, Figure 1A is still misleading as to the nature of the workflow. It suggests that you have 45 separate samples for each tissue or origin that were processed to RNA, when, in fact, you had 15 separate samples with RNA extracted and then you split the RNA into three separate libraries. Please change this workflow to reflect what was actually done. Change the yellow “45” to “15.” Change “225 samples” to “75 samples” and then alter the Figure to indicate that this was then used to generate technical replicates.

Done. This region of Fig 1 now looks as follows

Where the yellow text now shows the exact number of samples for each cancer type. These amount to 75 samples altogether. Each of the 75 was split into 3 replicates and 225 (75*3) libraries were prepared for sequencing

For Figure 3 A-C, please change the text over the yellow boxes (Module 2 for melanoma) to black to make it legible.

Done

For Figure 5, is there any way to color code the p-value column? Toward the paper’s argument that unique signatures are activated in each cancer, multiple of the signatures associated with module 3 and module 4 have much lower p-values than those associated with multiple cancer types.

As per the suggestion of the reviewer we color coded the *p* value column in Figure 5 with the highest *p* values in red transitioning to the lowest *p* values in white. We modified the **Fig** 5 caption to reflect this fact. With this color coding in place one can now easily see that most of the annotations with the lowest *p* values occur for those associated with one module/cancer only. This is particularly true for annotations associated with modules 3 and 4 as the reviewer has observed.

On page 9 it appears there is an erroneous line that says “Fig 10. Continued”

Removed.

On page 10 in the second paragraph of the discussion, the second line currently reads “cancer types and applicable…” and should be replaced with “cancer types and are applicable…”

Changed according to the reviewer’s suggestion.

Reviewer #7: The authors analyzed RNA-Seq data obtained from 225 tumor and normal canine samples and compared it to published human transcriptomic data to identify cancer-type-specific gene co-expression modules that are conserved between the two species. They then functionally annotated the modules to gain insights into biological mechanisms.

Major Comments

Novelty/originality of work
1. Dataset
All but the internal canine data has been already published.

The internal data has also now been published. It can be found at the NCI’s Integrated Canine Data Commons (ICDC) at <https://caninecommons.cancer.gov>

2. Methods
Authors use a straight-forward approach that averages gene expression counts per gene list per condition using standard R packages. It is not clear to me how this is superior to other widely-used approaches for gene expression analysis such as WGCNA.

We are not claiming that the method is superior, or even novel to other widely used methods. What is true, however, is that the combination of elements (access to canine samples, consideration of gene aggregates, analyzing multiple cancer data sets together and efforts to move away from murine models) is not common (especially access to the samples).

We added wording to this effect in the fourth paragraph of the introduction.

In addition, we removed the second to last paragraph of the discussion:

“Often, the top list of differentially expressed genes is submitted for annotation and co-expression may not be considered up-front. Genes chosen this way are more likely to be uncorrelated, expression wise, and considering them together can significantly reduce the number of biologically interpretable annotations found.”

Which may be interpreted as a claim of superiority of our methods relative to others.

3. Biology
The authors’ observations are consistent with the work done by multiple other teams. However, what exactly are the novel targets that this analysis has discovered?

**a.** There are novel targets, however the novelty of the targets was not properly elaborated/discussed in the manuscript. To address this issue, we added the following wording to paragraph 6 of the introduction:

” Many of the genes in these modules have little evidence of being targeted with drugs for the cancers studied. These genes, therefore, are good candidates for future target and/or drug discovery efforts. Annotations associated with these genes (*31, 32*) revealed some common, but mostly distinct biological mechanisms for each cancer (**Fig** 1I). Most of these mechanisms are known, but they provide validation for the co-expression modules, and biomarker genes selected therefrom”

**b**. We changed the title of the last section of the methods to:

**Biomarker link to literature and target novelty**

We added new material in this section to describe our protocol for determining target novelty. We basically use a combination of information from Pharos and CTD to determine how much information there is on chemistries active against the targets in question. Targets that are not associated with active chemistries are deemed underexplored with respect to drug discovery and so are labeled as novel targets.

**c.** We added the following section to the results:

**Many biomarkers selected from the primary modules of each cancer are underexplored as drug targets.**

In this section we describe use of our novelty protocol to assess our biomarker genes and we present the results of that assessment. In **Figs** 6-10 we added a column B called “IDG class” that shows the Pharos classification in terms of the amount of drug information associated with the targets. We highlighted in yellow in **Figs** 6-11 of the manuscript and in supplementary file **S7-annotations_gene_detail.xlsx** those targets deemed to be the most novel. Targets for which nothing is known, (Tdark IDG class) are further highlighted in maroon. Note that these highlighted targets are not currently associated with small molecule or drug activities.

**d.** We added material to the discussion starting with the third paragraph of page 18, that covers our concept of target novelty, with some illustrative examples.

**e.** We overlayed onto Fig 11 the target novelty status. Genes appended with one asterisk are “Tbio” targets, those appended with two asterisks are “Tdark”

Methodology
1. Model validation to human data.
Given that the human dataset used here for validation of the modules consisted of the same 5 cancer types, the specificity of the modules to these cancer types needs to be further explored. It is crucial that the validation dataset is expanded by other cancer types to prove that these cancer-type-specific modules (M1, M3 etc) won’t match to something unexpected.

**a.** Agreed. To address this point, we searched the Genomic Data Commons Data Portal (GDC) to find a larger human data set associated with treatment naïve patient samples from a variety of cancers other than the ones studied in this work. We found one, in the project titled, “NCI Cancer Model Development for the Human Cancer Model Initiative (HCMI-CMDC)”. There are 780 data files associated with this project.

**b.** We added material to the methods section titled “Model validation using external data” to show how we handled this data. In the end, we chose 53 read count files associated with tumors from patients with cancer of the small intestine (Adenocarcinoma, NOS (Not Otherwise Specified)), Brain cancer (Glioblastoma), colon cancer (Adenocarcinoma, NOS and Tubulovillous adenoma, NOS), esophageal cancer (Adenocarcinoma, NOS), bile duct cancer (Adenocarcinoma, NOS and Cholangiocarcinoma), pancreatic cancer (infiltrating duct carcinoma), and rectal cancer (Adenocarcinoma, NOS).

We also changed our random sub-sample cross validation method to ensure that the number of positive cases for tests of our classification models were the same for all cancers. This removes any ambiguity when interpreting ROCS results.

**c.** We added a new supplementary Fig S4 that gives the details of the new data added.

**d**: This new external data was seeded into the external data already collected from NCBI-GEO for human pulmonary carcinoma, melanoma, osteosarcoma, B- and T-cell lymphoma. Then we implemented our cross-validation procedure, generating ROC curves and associated statistics (AUC, Sensitivity, Specificity). **Fig** 4 has been augmented with these new results (Figure 4G and H).

**e**. In the results section, we added material to last paragraph of page 6, first paragraph of page 7 that describes the results

**3e**. In the discussion section we added material to page 17, 18 that further elaborates on the new results.

Minor points here: were all human data treatment naïve? Why did the classification model perform better on human data than on external canine data (Figure 4)?

Human data was for treatment naïve patients and this note has been added to the text.

Regarding the better performance of the classification model on human data: This could have multiple causes. The thing that would most dramatically affect the ROCS statistics is the distribution of expression values in training and test sets (all else being equal). With this idea in mind, we took a closer look at our methodology. We perform random sub-sample cross validation to test our models. When testing a model, we pool all data together, then split it evenly into training and test sets, requiring that the number of positive cases (for the cancer we are focusing on) is the same in training and test sets. However, the data sets for each cancer type are of different size. So, although the training and test set sizes remain identical (because we pool all data then split it), the number of positive examples in training and test will be different for each cancer type and so will the ratio of positives (different for each cancer) to negatives (fixed). This will lead to ambiguities in interpretation of the ROCs results. To remove any ambiguities, we required also that these ratios be the same for all cancer types. Therefore, the number of positives in training and test sets is determined by that data set with the least amount of data associated with it. This turns out to be 18 positives split into 9 for training and 9 for test, e.g., GEO data set for lung adenocarcinoma, **Fig** S3. If a particular cancer had more than 18 examples, we selected 18 at random, and threw out the rest of the data for that cancer type. Then with the remaining data we performed 100 trials of random subsample cross validation. So, we effectively sample the data splits, but only on a subset of the data we have access to.

This is what we did before, and generated results that were better for human than dog.

This issue, however, is that we performed 100 trials of random subsample cross validation on only a single selection of 18 positives associated with each cancer. What we should have done was perform multiple random selections of 18 samples for each cancer type and then for each 100 trials of random sub-sample cross validation. The multiple random selections of 18, samples all of the data available to us, the 100 rounds of cross-validation for each selected subset of 18, effectively samples the data splits (9 training, 9 test).

In the end we selected at random 18 samples for each cancer type from the available data and combined these together to from our data subset. Then for the given data subset we ran 200 iterations of random subsample cross validation to gather the rocs statistics. We repeated this procedure 20 times to converge the ROCS statistics (4000 trials). We performed this procedure for the canine external tests and human external tests. These converged results are shown in Figure 4. This new procedure effectively utilizes all data available to us for the cancer types studied.

The results are not much different than previous, but they do show the following trend in ROCS statistics from best to worse depending on the data sets used:

Canine internal (AUC = 0.99), canine external (AUC = 0.97), human external (AUC = 0.95), human external augmented with cancer types other than MEL, PULM, OSA, BLSA, and TLSA (AUC = 0.91).

Note that we pegged the specificity at ~0.7 to keep the sensitivity values roughly 0.7 or higher.

We do not know if this ordering would hold up with different selections of canine and human data pulled from public resources. The problem is that there are simply too many unknowns to control for, e.g., different experimental procedures in different labs, different informatics approaches etc. But at present, at least, we have completely under control sampling for a given data set. It is remarkable that the ROCS statistics hold up (see discussion above), even when we add data pertaining to cancer types other than those for which we built the models. The selective activation of the primary modules for the specific cancer types studied here (MEL, PULM, OSA, BLSA, and TLSA) among 10 -15 cancer types included (Figure 4 g, h) indicates that the biomarkers from the primary modules of MEL, PULM, OSA, BLSA, and TLSA warrant further study.

We made multiple modifications to the manuscript to reflect changes to the methodology discussed above. See modifications made to the methods section titled “Model validation using external data”, the results section titled “The nearest neighbor models successfully classified …” and the discussion section , pages 17 and 18.

2. Primary modules selection.
“After the profiles were computed, the primary module of each cancer was determined as the highest up- regulated module, statistically different between cancer and normal states.”
This makes me think that over-expression is the only mechanism of cancerogenesis that was considered in this study. Authors should explain why they didn’t look at the opposite trend, most down-regulated modules. For example, if a module consists of tumor suppressors, down-regulation might be a possible mechanism of tumorigenesis.

We did look at both positive and negative trends, but in the end only the positive trends were significant with this data. The explanation is as follows:

The only way to obtain up and down-regulated genes is to compare the gene read counts between cancer and normal control, e. g. Log2(FC). In our case the normal samples (except for PULM) are not from the same tissue type as the cancer samples, so a log2(FC) of cancer samples relative to the normal samples does not make sense. Rather, we looked at Z scaled read count values . Our Z-scaled read count data has median value of -0.18, and 0%, 25%, 50%, 75% and 100% quantiles = -0.75, -0.36, -0.18, 0.05, and 49.29, respectively. The Figure below shows the Z matrix gene expression distribution values in black, and primary module gene expression distributions in various other colors:

Range = -0.75 to +49.30

The expression values essentially start at 0 and are splayed in a positive direction. The two vertical grey lines at +1.5 and -1.5 mark the cut-off for statistically significant gene expression (Z scores) relative to background. Note that all the significant genes have positive Z scores.

These Z scaled read counts, are essentially a measure of absolute activation with 0 being the reference and are read counts processed into a form and range amenable to module calculations. The positive splayed values of the Z matrix with minimum near 0 reflect the properties of the original read count matrix, from which it was derived (minimum value = 0, all counts positive), We added wording to paragraph 2 of the results to make these points. We also added the following verbiage at the end of the third paragraph of the discussion:

“For PULM, it may be possible to deconvolute tissue specific transcription from cancer specific transcription based on the *p* values for comparison of gene expression in tumors to their valid normal control gene expression. For the other tumor types, this deconvolution based on *p* values is not possible because the normal samples come from a different tissue source than the tumors. We may, however, use the Z values in these cases to determine if the genes are significantly expressed relative to background by considering only Z values ≥ 1.5 (~95% confidence level for Z scores). Using these genes, it then may be possible, to use gene annotations and inclusion in COSMIC (*38*) and OncoKB (*39*) to decide if module genes are related to cancer and not just tissue specific expression..”

In the discussion on PULM (page 16) where we do have valid normal samples, we see that there are genes that are downregulated relative to normal. Two of these genes, NAPSA (PULM activation = 2.73, Normal activation = 4.43, *p* = 0.45) and ADGRF5 (PULM activation = 2.73, Normal activation = 3.31, *p* = 0.85) are mentioned in the text. Though these genes are down-regulated relative to normal controls, their reduced expression is not statistically significant. Other genes with PULM activation > 1.5 but are down-regulated relative to their normal controls are MAN1A1, SPTBN1, TMBIM6, METTL7A, NAPSA, ADGR5, ALCAM, and NPNT. However, *p* values for comparison to normal controls are all > 0.05 and not significant. The only genes with activation > 1.5 with significant expression relative to normal are CD44, FAT1, SDC1, and MYOF (second paragraph page 16) but these genes are all up-regulated relative to normal.

So, in the current study there are simply no genes that can be termed “down-regulated” , either because we are using Z scores (with minimum 0 and splayed positively) where activation is referenced to a zero background in cases where no valid normal exists (MEL, OSA, BLSA, TLSA), or in cases where valid normal samples exist (PULM), all the genes with statistically significant activation relative to normal are up-regulated.

To further reduce the confusion, in the text the term “up-regulated” has be replaced with the term “significantly expressed”.

3. PCA analysis.
The PCA plot (Figure 3) shows only PC1-PC3. Perhaps I missed it, but it would be interesting to see what feature PC2 corresponds to.

A PC1-PC2 plot is shown below:

We did not choose to show this plot, because the separation of tumor samples for OSA, PULM, and MEL is not as dramatic as it is for the PC1-PC3 plot shown in Figure 3E. In the plot above most of the variation along PC2 is in the BLSA and TLSA data. The OSA, PULM, and MEL data is a jumble in the upper left corner. Since there is already good separation of BLSA and TLSA along PC1, we decided to look at PC3 along the horizontal axis to see if there was more variation in the OSA, PULM, and MEL data. In the plot of Figure 3E, we see that along PC3, the variation due to BLSA and TLSA is essential reduced to zero, however the variation contribution due to the OSA, PULM, and MEL data is quite large. This variation serves to separate out the OSA, PULM, and MEL points to produce the nice plot of Figure 3E.

Also, from my experience, sample purity is often a confounding factor in such studies. So, I would like to see scatter plots for purity (or tumor to stroma cell ratios) ~ PC1/2/3, to make sure it is not the case here.

This question of sample purity is important, so we put our response into a supplementary file called S8-Tumor_stroma_breed-purity.docx. Please read it for our response.

4. Normal tissue.
The “… B and T cell lymphoma normal tissues as skin with an admixture of adipose tissue, the osteosarcoma and melanoma normal samples were mainly skin with an admixture of muscle tissue, the pulmonary carcinoma normal samples were mainly lung with an admixture of muscle tissue.”
Gene expression is known to be highly tissue specific. Given that for all but one cancer types the normal sample was not represented by the same tissue type as the tumor sample, is it sensible to select cancer-specific modules by comparing activation profiles between tumor and not-matching control samples?

We totally agree with the reviewer. Please see discussion above in the section titled “2. Primary modules selection” We believe that this answers the question.

5. Dog breeds.
The dataset used in this study contains RNA-Seq data on multiple dog breeds. In contrast to human genetics, purebred dogs are known to have high levels of homozygosity, which is another reason why transcriptomic studies of canine cancers are so intriguing. The authors have breed information for almost all samples in S1. In my opinion, these data should be used to stratify transcriptomic data prior to gene co-expression analysis. At least the authors should check how much variance it accounts for. Is it PC2?

This question on dog breeds is important, so we put our response into a supplementary file called S8-Tumor_stroma_breed-purity.docx. Please read it for our response.

6. Modules annotations.
Authors used DAVID tool for functional annotations. To increase clinical relevance of this study, it would be interesting to overlap the modules with known cancer genes from COSMIC or OncoKB.

We have computed these overlaps as the reviewer suggests. To briefly summarize:

For PULM, all module 1 genes were associated with PULM in COSMIC and 4 of these were also found in OncoKB. For MEL, all module 2 genes were associated with MEL in COSMIC and two of these were also found in OncoKB. For OSA module 3 genes, eight were associated with OSA in COSMIC, and two were found in OncoKB. For BLSA module 4 genes, three were associated with B-Cell lymphoma in COSMIC and 3 genes were found in OncoKB. For T-cell lymphoma six genes were found in COSMIC and 13 were found in OncoKB.

We have overlayed these results, along with the new target novelty results onto Fig 11. In Fig 11, genes colored pink are overlaps between COSMIC and our gene modules, genes in thick boxes are overlaps between OncoKB and our module genes, and genes colored pink and boxed are those module genes that are present in both COSMIC and OncoKB. We also added drug target novelty information. Genes appended with one asterisk are Tbio targets according to Pharos (they have associated gene ontology terms or a confirmed OMIM phenotype) and have no known active small molecule chemistries against them. Genes appended with two asterisks are Dark targets according to Pharos (unstudied targets). See the methods section for more information regarding Pharos designations.

We added a paragraph (last paragraph, page 10) that presents details of the overlap between our module genes and both COSMIC and OnkoKB.

And in the discussion, page 18 we added a detailed discussion pertaining to the use of PHAROS, COSMIC, OnkoKB, and **Fig** 11 to generate high priority lists of novel drug target genes.

Minor Comments

Text
1. Page 3.
“Genes in a co- expression module are of interest because such genes are thought to members of the same biological pathways or part of the same transcriptional regulatory program.” --> “Are thought to be members”

Fixed

2. Page 4
“Though this work is strictly a study on gene expression, the annotations provide a means for linking the most highly expressed genes for each cancer type studied with mutational events known to drive the cancers. “ --> Did the authors actually check that? If not, maybe it is better to move this to Discussion.

We did check this (see, for example, last paragraph page 13) but we agree with the reviewer that these comments are better moved to the discussion. This sentence was removed from the intro, slightly modified, and placed in the first paragraph of page 13 in the Discussion.

“Each RNA sample from the single tumor biopsy was divided to make 3 libraries for RNA-Seq. “ --> Please explain the rationale.

The rationale is explained below and added to the methods section in the subsection titled RNA-Seq Experiments

“Each RNA sample (75 altogether) from the single tumor biopsy was divided into 3 replicates. These replicates were used to make 3 cDNA libraries for sequencing. Each of the 3 libraries was loaded onto 3 different lanes on the flow cell that goes into the sequencer. The replicates allow for estimation of within sample variability of gene expression which allows us to then make inferences between samples of the same cancer type or between samples of different cancer types (*86*).”

3. Page 6.
“Each cancer exhibits a unique module expression profile.” --> This phrase sounds out of context and really stands out, please consider rephrasing.

This has been changed to

“Each cancer exhibits significant activation in a different module.”

4. Methods
“See supplementary Fig S1 and supplementary file S1 for details regarding the tumor sample histologies and location of normal samples.” – I did not find any info on location of normal samples in S1.

We created a supplementary Figure S2 with the normal sample details and we changed the above sentence to:

See supplementary **Fig** S1 and supplementary file **S1-Biospecimens.xlsx** for details regarding the tumor sample histologies and supplementary **Fig** S2 regarding normal sample histologies.

Figures
1. Figure 1A.
Explain clearer where the 225 samples come from and/or explain in the text how many tumor and normal samples were collected per animal.
“We prepared 225 samples for RNA-Seq analysis: 12 tumor samples and 3 normal samples in triplicate for each cancer type (Fig 1B)”. --> please, specify these are technical replicates.

In the introductory discussion concerning Figure 1B we created the following text (last paragraph page 3)”

We prepared 75 samples for RNA-Seq analysis (**Fig** 1A). Each samples was split into 3 replicates to make 3 cDNA libraries for sequencing analysis (**Fig** 1B). All told 225 libraries were sequenced.

In the results section paragraph 1 we add a little more detail:

“Each RNA sample from the single tumor biopsy was divided into 3 replicates. Each replicate was used to make 3 cDNA libraries for sequencing. (*33*) (*34*) Each of the 3 libraries was loaded onto 3 different lanes on the flow cell that goes into the sequencer. 225 libraries in all were sequenced”.

And in the methods titled **RNA-Seq Experiments** we give the most detail:

“Each RNA sample (75 altogether) from the single tumor biopsy was divided into 3 replicates and each replicate was used to make 3 cDNA libraries for sequencing. Each of the 3 libraries was loaded onto 3 different lanes on the flow cell that goes into the sequencer. 225 libraries altogether were sequenced. The replicates allow for estimation of within sample variability of gene expression which allows us to then make inferences between samples of the same cancer type or between samples of different cancer types (*84*)”

It should be clear now were the 225 samples come from.

2.

Figure 1B.
Seems unnecessary to show how RNA-Seq works in this dry lab paper. Also, since there’s no explanation in the text, better to leave it out.

As seen above at the reviewer’s request, we have added more explanation to the text regarding the samples, library construction, replicates, and flow cell lanes to ensure that the reader knows exactly what is going on with the samples, replicates, libraries, and what is sequenced. Because of these added details we decided to leave Figure 1B as is.

3. Figure2.
As it is now, there are multiple genes (i.e. PKNOX2 from M2) that don’t change the expression between tumor and normal and hence are not informative. Consider showing a heatmap with only “biomarkers” (“genes with activation > 1.5”) and move the current version to Supplementary Figures. Also, if possible, use a more standard heatmap color scheme (red/blue).

Done, see new Figure 2. Even when including all genes in the modules the Figure is not that much different, so we don’t include it in supplementary figures.

4. Figure 3A-C.
It will help the reader a lot if you order cancer types in this table according to modules number (i.e. PULM --> MEL --> OSA etc)

Done, see new Figure 3.
5. Figure 3D.
Why so many outliers for M4 in BLSA normal samples?

We do not know why there are a lot of outlier genes, this could be do to a lot of factors. However, some of these outliers will certainly be of interest as important genes related to the cancers they are associated with while others may be artifacts related to how the experiments were done, human error, etc. We believe that these outliers, to a large extent are important genes. Our logic is as follows:

First, we increased the y range in Fig 3D so that all outliers can be viewed. The new Fig 3D shows that all modules contain outlier genes, not just M4 for BLSA samples. In the Figure below

we show the gene expression distributions for the Z matrix (all genes, all samples, color = black) and the module subsets of the Z matrix (primary module genes and cancer samples they are associated with) in various other colors. The cut-off for significantly expressed genes (relative to background) is shown as two vertical grey bars at +1.5 and -1.5. The Z matrix gene expression distribution is not normal, with median value of -0.18, and 1’st, 2’nd, and 3’rd quantiles = -0.36, -0.18, and 0.05, respectively. In the simplest case, we define an outlier as any point of data that lies over 1.5 IQRs below the first quantile, or 1.5 IQRs above the third quantile in the data set. The inter-quantile range (IQR) = Q3 -Q1 = 0.41. Therefore, outliers are defined as any gene with Z score < -0.975 or > 0.665. Our cut-off for significant gene expression relative to background is ±1.5. so, all our statistically significant genes (relative to background) are also outliers (when we consider the Z matrix distribution), and we assume that these outlier observations are of interest as genes important to the cancers.

For the individual primary module genes and their average activation across the cancer samples that they are associated with, the distributions are right skewed (not normal) and the cut-offs for outliers vary. Looking at the Box Plot distributions of Figure 3D we can determine the outlier cutoffs of 5.04, 3.87, 2.18, 2.22, and 2.36 for BLSA, TLSA, OSA, MEL, and PULM, respectively. There are no outliers in the negative direction. Looking at Figures 6-10 of the text, the outlier cut-off values, and the value for statistical significance (±1.5) we can determine the following:

BLSA, 24 significant genes, 10 outliers

TLSA, 48 significant genes, 10 outlier genes

OSA 21 significant genes , 12 outlier genes

MEL 27 significant genes, 16 outlier genes

PULM 32 significant genes, 23 outlier genes

To put this in perspective if we generate the same number of points (at random) from a normal distribution than we have genes in the primary modules associated with each of our cancers then the number of outliers for each of our cancer types (generated by chance alone and not to error) would be:

BLSA outliers = 5

TLSA outliers = 7

OSA outliers = 7

MEL outliers = 13

PULM outliers = 14

These numbers coming from a normal distribution (and due to chance alone) are close to the numbers coming out of our skewed distributions for gene expression. Therefore, we believe that outliers are not confounding factors in our work. Other evidence that suggests that the outliers are not an issue can be found in the case of Melanoma (Fig 7). The most highly expressed gene (and also the largest outlier) in module 2 associated with melanoma is GPNMB (Z = 18.59). However, we know that GPNMB is an important melanoma gene. See discussion on page 15 relating to the importance of GPNMB in melanoma.

There is no precise definition of “outlier gene”, even for a normal distribution. Ultimately it depends on an observer being “surprised”, or not, by a particular result. Given the above considerations, we don’t find any reason to be surprised by any of the highly expressed genes in our modules.

6. Figures 5-10.
These tables should be replaced by a plot that is easier to understand, for example, boxplots showing CTD values per module.

We agree with the reviewer that the data in these tables can always be made more understandable. These tables contain a lot of information and recasting to improve understanding will take far longer time than we have at present to get the paper back to the journal. For these reasons we have left Figs 5-11 as is.

**Have the authors made all data and (if applicable) computational code underlying the findings in their manuscript fully available?**
The [PLOS Data policy](https://journals.plos.org/ploscompbiol/s/materials-and-software-sharing) requires authors to make all data and code underlying the findings described in their manuscript fully available without restriction, with rare exception (please refer to the Data Availability Statement in the manuscript PDF file). The data and code should be provided as part of the manuscript or its supporting information, or deposited to a public repository. For example, in addition to summary statistics, the data points behind means, medians and variance measures should be available. If there are restrictions on publicly sharing data or code —e.g. participant privacy or use of data from a third party—those must be specified.

Reviewer #2: Yes

Reviewer #4: Yes

Reviewer #5: Yes

Reviewer #6: Yes

Reviewer #7: Yes

PLOS authors have the option to publish the peer review history of their article ([what does this mean?](https://journals.plos.org/ploscompbiol/s/editorial-and-peer-review-process#loc-peer-review-history)). If published, this will include your full peer review and any attached files.


If you choose “no”, your identity will remain anonymous but your review may still be made public.


**Do you want your identity to be public for this peer review?** For information about this choice, including consent withdrawal, please see our [Privacy Policy](https://www.plos.org/privacy-policy).

Reviewer #2: No

Reviewer #4: No

Reviewer #5: No

Reviewer #6: No

Reviewer #7: No

Figure Files:

While revising your submission, please upload your figure files to the Preflight Analysis and Conversion Engine (PACE) digital diagnostic tool, <https://pacev2.apexcovantage.com>. PACE helps ensure that figures meet PLOS requirements. To use PACE, you must first register as a user. Then, login and navigate to the UPLOAD tab, where you will find detailed instructions on how to use the tool. If you encounter any issues or have any questions when using PACE, please email us at [figures@plos.org](mailto:figures@plos.org).

Data Requirements:

Please note that, as a condition of publication, PLOS' data policy requires that you make available all data used to draw the conclusions outlined in your manuscript. Data must be deposited in an appropriate repository, included within the body of the manuscript, or uploaded as supporting information. This includes all numerical values that were used to generate graphs, histograms etc.. For an example in PLOS Biology see here: <http://www.plosbiology.org/article/info%3Adoi%2F10.1371%2Fjournal.pbio.1001908#s5>.

Reproducibility:

To enhance the reproducibility of your results, we recommend that you deposit your laboratory protocols in protocols.io, where a protocol can be assigned its own identifier (DOI) such that it can be cited independently in the future. Additionally, PLOS ONE offers an option to publish peer-reviewed clinical study protocols. Read more information on sharing protocols at <https://plos.org/protocols?utm_medium=editorial-email&utm_source=authorletters&utm_campaign=protocols>

References:

Review your reference list to ensure that it is complete and correct. If you have cited papers that have been retracted, please include the rationale for doing so in the manuscript text, or remove these references and replace them with relevant current references. Any changes to the reference list should be mentioned in the rebuttal letter that accompanies your revised manuscript.

If you need to cite a retracted article, indicate the article’s retracted status in the References list and also include a citation and full reference for the retraction notice.
