# Supplementary material for: Transcriptomic profiling in canines and humans reveals cancer specific gene modules and biological mechanisms common to both species
Source: PLoS Comput Biol. 2021 Sep 27;17(9):e1009450. doi: 10.1371/journal.pcbi.1009450 (PMC8523068; doi:10.1371/journal.pcbi.1009450)
Supplement: Attachment — Submitted filename: GT_response_v3_D-21-00257.docx [file pcbi.1009450.s016.docx]

Dear Dr. Gallo,

Please see below for original text that you sent containing instructions for resubmitting a revised manuscript and the reviewer responses to the manuscript. Our responses to the reviewer’s comments are shown in red and the revised manuscript (attached with this letter) has been color coded appropriately to reflect changes suggested by the reviewers. Also attached is a clean version of the manuscript).

If there is anything else that you need, please let me know.

Sincerely

Gregory J. Tawa

**___________________________________________________**

**Gregory J. Tawa**

**Modeling and Informatics Lead**

**NIH NCATS (National Center for Advancing Translational Sciences)**

**Therapeutic Development Branch**

**DIVISION OF PRE-CLINICAL INNOVATION (HN92)**

**9800 MEDICAL CENTER DR-BLDG C**

**ROCKVILLE MD 20850**

| **Office phone: 301-827-7177**  **Mobile: 240-550-0749**  **Email:Gregory.tawa@nih.govv** |  |
| --- | --- |

Dear Dr. Tawa,

Thank you very much for submitting your manuscript "Transcriptomic profiling in canines and humans reveals cancer specific gene modules and biological mechanisms common to both species" for consideration at PLOS Computational Biology. As with all papers reviewed by the journal, your manuscript was reviewed by members of the editorial board and by several independent reviewers.

The paper has been greatly improved and can be accepted once some minor comments from one of the reviewers can be addressed.

Please prepare and submit your revised manuscript within 30 days. If you anticipate any delay, please let us know the expected resubmission date by replying to this email.

When you are ready to resubmit, please upload the following:

[1] A letter containing a detailed list of your responses to all review comments, and a description of the changes you have made in the manuscript. Please note while forming your response, if your article is accepted, you may have the opportunity to make the peer review history publicly available. The record will include editor decision letters (with reviews) and your responses to reviewer comments. If eligible, we will contact you to opt in or out

[2] Two versions of the revised manuscript: one with either highlights or tracked changes denoting where the text has been changed; the other a clean version (uploaded as the manuscript file).

Important additional instructions are given below your reviewer comments.

Thank you again for your submission to our journal. We hope that our editorial process has been constructive so far, and we welcome your feedback at any time. Please don't hesitate to contact us if you have any questions or comments.

Sincerely,

James Gallo

Associate Editor

PLOS Computational Biology

Florian Markowetz

Deputy Editor

PLOS Computational Biology

***********************

A link appears below if there are any accompanying review attachments. If you believe any reviews to be missing, please contact [ploscompbiol@plos.org](mailto:ploscompbiol@plos.org) immediately:

Reviewer's Responses to Questions

**Comments to the Authors:
Please note here if the review is uploaded as an attachment.**

Reviewer #5: The authors have addressed all my concerns. The manuscript is ready for publication.

Reviewer #6: The authors have done a very thorough job of responding to the reviewer comments through multiple rounds of review.

Reviewer #7: The authors did a lot of work on addressing my and other reviewers’ comments. In my opinion, this manuscript has improved dramatically since the last submission. The authors improved their methodology using more appropriate subsampling strategy and added an important analysis piece on validating their results on a larger human dataset on naïve tumors of various cancer types other than the ones studied in this work. Another important type of the analysis that was performed was a comprehensive dog breed and tumor purity analysis. And, lastly, the authors annotated their gene modules with COSMIC and OncoKB. In my opinion, the large overlap with COSMIC/OncoKB genes has raised the relevance of this work.
I would also like to acknowledge how the discussion section has improved and expanded. In particular, by adding the discussion on the novelty of the targets.
Moreover, the authors have published their dataset.

Taken together, I recommend this manuscript for publishing after fixing some of the minor points that I am listing below.

Text

1. Page 3 starting with “This complexity may involve” a typo and/pr --> and/or

Correction made
2. Page 4 starting with “The work presented herein”. As “relevance of canine to human cancer” seems confusing, perhaps, use “relevance of canine-to-human cancer studies” instead?

Correction made
3. Page 5. Capitalize P-values at the beginning of the sentence (“P-values associated.. “)

Correction made
4. Page 11 starting with “Most of these targets”” “are involved the process” is missing “in”

Correction made

Figures

1. Figure 2.
a. Since red/green color scheme is not colorblind-friendly, consider changing to the previously suggested red/blue.

Done
b. Instead of showing g1,g2,g3 for each module, show some of the real gene names that you mention in the discussion section.

Done
c. For the consistency, please reorder as in Figure 3 PULM>MEL>OSA etc

Done
2. Figure 3.
a. Please add module size to the x axis labels as BLSA (M4) N=…genes

**Done**
b. Please label some at least some of the “outlier observations that are of interest as genes important ton cancers”. These are indeed the most interesting candidates, and the readers will benefit from knowing how the corresponding expression levels are distributed.

Done
c. Y axis label. Please add units (Z scores)

**Done**

**Have the authors made all data and (if applicable) computational code underlying the findings in their manuscript fully available?**
The [PLOS Data policy](https://journals.plos.org/ploscompbiol/s/materials-and-software-sharing) requires authors to make all data and code underlying the findings described in their manuscript fully available without restriction, with rare exception (please refer to the Data Availability Statement in the manuscript PDF file). The data and code should be provided as part of the manuscript or its supporting information, or deposited to a public repository. For example, in addition to summary statistics, the data points behind means, medians and variance measures should be available. If there are restrictions on publicly sharing data or code —e.g. participant privacy or use of data from a third party—those must be specified.

Reviewer #5: Yes

Reviewer #6: Yes

Reviewer #7: None

PLOS authors have the option to publish the peer review history of their article ([what does this mean?](https://journals.plos.org/ploscompbiol/s/editorial-and-peer-review-process#loc-peer-review-history)). If published, this will include your full peer review and any attached files.


If you choose “no”, your identity will remain anonymous but your review may still be made public.


**Do you want your identity to be public for this peer review?** For information about this choice, including consent withdrawal, please see our [Privacy Policy](https://www.plos.org/privacy-policy).

Reviewer #5: No

Reviewer #6: No

Reviewer #7: No

Figure Files:

While revising your submission, please upload your figure files to the Preflight Analysis and Conversion Engine (PACE) digital diagnostic tool, <https://pacev2.apexcovantage.com>. PACE helps ensure that figures meet PLOS requirements. To use PACE, you must first register as a user. Then, login and navigate to the UPLOAD tab, where you will find detailed instructions on how to use the tool. If you encounter any issues or have any questions when using PACE, please email us at [figures@plos.org](mailto:figures@plos.org).

Data Requirements:

Please note that, as a condition of publication, PLOS' data policy requires that you make available all data used to draw the conclusions outlined in your manuscript. Data must be deposited in an appropriate repository, included within the body of the manuscript, or uploaded as supporting information. This includes all numerical values that were used to generate graphs, histograms etc.. For an example in PLOS Biology see here: <http://www.plosbiology.org/article/info%3Adoi%2F10.1371%2Fjournal.pbio.1001908#s5>.

Reproducibility:

To enhance the reproducibility of your results, we recommend that you deposit your laboratory protocols in protocols.io, where a protocol can be assigned its own identifier (DOI) such that it can be cited independently in the future. Additionally, PLOS ONE offers an option to publish peer-reviewed clinical study protocols. Read more information on sharing protocols at <https://plos.org/protocols?utm_medium=editorial-email&utm_source=authorletters&utm_campaign=protocols>

References:

Review your reference list to ensure that it is complete and correct. If you have cited papers that have been retracted, please include the rationale for doing so in the manuscript text, or remove these references and replace them with relevant current references. Any changes to the reference list should be mentioned in the rebuttal letter that accompanies your revised manuscript.

If you need to cite a retracted article, indicate the article’s retracted status in the References list and also include a citation and full reference for the retraction notice.
